# Prevalence and potential risk factors associated with idiopathic epilepsy in Golden Retrievers

**DOI:** 10.1093/jvimsj/aalag230

**Published:** 2026-10-10

**Authors:** Maria Winnerby, Cecilia Rohdin, Erling Strandberg, Hanne Gredal

**Affiliations:** AniCura Specialistdjursjukhuset Albano, Rinkebyvägen 21B, 182 36 Danderyd, Sweden; AniCura Specialistdjursjukhuset Albano, Rinkebyvägen 21B, 182 36 Danderyd, Sweden; Department of Animal Biosciences, Swedish University of Agricultural Sciences, 750 07 Uppsala, Sweden; University Hospital for Companion Animals, Department of Veterinary Clinical Sciences, University of Copenhagen, Dyrlægevej 16, 1870 Frederiksberg C, Denmark

**Keywords:** cereal-free diet, cluster seizure, gastrointestinal disease, neutered status, risk factors

## Abstract

**Background:**

Idiopathic epilepsy (IE) in Golden Retrievers (GRs) has a suspected genetic basis with several endogenous risk factors potentially affecting the disease, including sex, breed subtypes, and chronic comorbidities. A subdivision into working (W-GRs) and show (S-GRs) Golden Retrievers presents a unique opportunity to investigate possible line-related risk factors.

**Hypothesis/Objectives:**

Define prevalence and investigate risk factors for IE in a population of Swedish GRs. We hypothesized a high prevalence of IE in GRs characterized by frequent cluster seizures, with the following representing potential risk factors for IE: subtype W-GR, male, and gastrointestinal or dermatologic signs or both.

**Animals:**

Twenty-nine client-owned GRs with IE and 714 controls.

**Methods:**

Prospective questionnaire-based study exploring the dog’s health status, living conditions, and possible seizures, including questions regarding seizure characteristics, treatments, and clinical outcomes in affected dogs. Data was analyzed using descriptive and inferential statistics and a logistic regression model was used to estimate the effect of potential risk factors on the probability of IE.

**Results:**

The prevalence of IE in GRs was 3.9%, with 48.2% presenting with cluster seizures. A significant difference in prevalence of IE between W-GRs and S-GRs was not found. Being male, showing signs of gastrointestinal disease, and cereal-free diets were associated with increased risk of IE.

**Conclusions and clinical importance:**

Golden retrievers have an increased risk of IE, often presenting with cluster seizures. The association between IE and gastrointestinal disease might lead to new therapeutic strategies, but further investigations are needed.

## Introduction

Idiopathic epilepsy (IE) is characterized by a predisposition to generate recurrent, unprovoked seizures with no identifiable cause.^1^ The prevalence of IE in dogs is estimated at 0.62%, but the condition exhibits considerable differences in prevalence and phenotypic heterogeneity across breeds.^2^^,^^3^ Seizure characteristics such as seizure manifestation, severity, frequency of cluster seizures (CS), frequency of status epilepticus (SE), and epilepsy-related mortality also differ across breeds.^2^^,^^4–9^ Several endogenous risk factors or comorbidities might accelerate seizure generation, including sex, breed, and chronic disease, and notably, stress might be an important precipitating factor for seizures in both humans and dogs with IE.^10–16^

Idiopathic epilepsy has a suspected genetic etiology in Golden Retrievers (GRs) and a suggested male predominance, but current prospective studies are lacking.^4^^,^^17^ The disease is predominantly characterized by generalized tonic–clonic seizures (GTCS) that might be difficult to treat.^18^

The GR ranks prominently among registered dog breeds in Sweden, with approximately 15 000 GRs registered in the Swedish Kennel Club. The breed is unofficially divided into 2 distinct subtypes: working Golden Retrievers (W-GRs) and show Golden Retrievers (S-GRs). Working Golden Retrievers are selectively bred for functional purposes and exhibit high energy levels, potentially making them more susceptible to stressors. This division provides a unique opportunity to investigate epilepsy and potential seizure triggers within the same breed.

In humans, patients with IE commonly exhibit medical comorbidities, including irritable bowel syndrome and allergies, conditions that are seen more often in GRs than in other breeds.^19–25^ These comorbidities might result from shared pathophysiological mechanisms such as stress or the epileptic state itself, or they might simply be an incidental co-occurrence.^19^^,^^20^ At present, evidence linking IE with gastrointestinal inflammatory diseases and allergies in dogs is limited, but the potential correlation warrants investigation.

Our prospective study aimed to describe the phenotype and seizure characteristics in Swedish GRs and to investigate possible risk factors and comorbidities related to breed subtype, sex, and concurrent medical conditions, including clinical signs of gastrointestinal and dermatologic disease. We hypothesized an increased prevalence of IE in the breed, compared with what is reported in the general dog population (>0.62%), and characterized by frequent cluster seizures and significant associations with gastrointestinal or dermatologic signs, subtype W-GR, and male as potential risk factors for IE.

## Materials and methods

Owners of Swedish GRs were invited to participate in a study evaluating epilepsy in the breed. Owners were asked to answer a questionnaire named “Health Questionnaire for Golden Retrievers”, hosted on the digital platform Netigate from September 2023 to December 2023. A complete description of the recruitment process is provided in Appendix.

The questionnaire (the questionnaire was originally provided in Swedish; an English version is provided in supplementary material) included 3 main sections concerning (1) signalment of the dog and living conditions, (2) general health status, and (3) seizure characteristics. The general health section included questions focusing on gastrointestinal and dermatologic signs.

Section 3 was only distributed to owners who had replied affirmatively to the main question (MQ) “Has your dog ever experienced a seizure?”. The seizure-related questions were adapted from a previous study.^8^

Golden Retrievers with or without epilepsy were eligible for inclusion if the owners had replied to the MQ and if the dogs were alive at the start of the study. The MQ was supplemented with a video of a GR experiencing a GTCS (Video 1). Golden Retrievers meeting the diagnostic criteria for IE following the International Veterinary Epilepsy Task Force (IVETF) guidelines (with the exception of urinalysis) were included in the IE group, and GRs showing no seizures were included in the control group.^1^ If the MQ was not answered, dogs had only had 1 seizure, the seizures did not fulfill the IVETF criteria for IE, or if the dogs were euthanized before the start of the study, they were excluded from the study.

Data unrelated to the objective of this study from 5 of the dogs in the IE group was also included in a previous study.^18^

Permission to access medical records was asked from owners of dogs with more than 1 seizure, both from the Anicura Sweden database and from local veterinarians. After consent, a European College of Veterinary Neurology resident (M.W.) collected data from these records, including clinical data to support fulfillment of criteria for a diagnosis of IE according to the IVETF guidelines.

### Statistical analysis

Descriptive statistical analyses were performed using a commercially available statistical software program (JMP Pro v. 11.2.0-17.2.0, Cary, NY, United States). Data were analyzed using descriptive and inferential statistics. Descriptive statistics were reported for continuous variables using mean (SD) for approximately normally-distributed values, and median (interquartile range [IQR]) for non-normally distributed data. We fitted a logistic regression model to estimate the effect of potential risk factors on the probability of IE. A detailed description of the statistical analysis is provided in Appendix.

## Results

The questionnaire yielded 1,252 responses. Of these, 758 owners responded to the MQ (Figure 1). Forty-four (5.8%) owners reported that their GR had experienced at least 1 seizure during its lifetime, 41 of these dogs had experienced more than 1 seizure. Twenty-nine were alive at the beginning of the study and fulfilled the tier I or II criteria for IE. The IE group included 20 male dogs (9 neutered; all before seizure onset) and 9 female dogs (3 neutered; 1 after seizure onset) (Table 1). Thirteen dogs were S-GRs, 12 were W-GRs, and 4 were of unspecified subtype. Fifteen of the 44 dogs with seizures were excluded because they did not fulfill the criteria for IE (Figure 1). The control group included 714 dogs with no history of seizures 338 were males (79 neutered) and 376 were females (69 neutered). Four hundred forty-three dogs were S-GRs, 246 were W-GRs, and 25 were of unspecified subtype.

**Figure 1:**
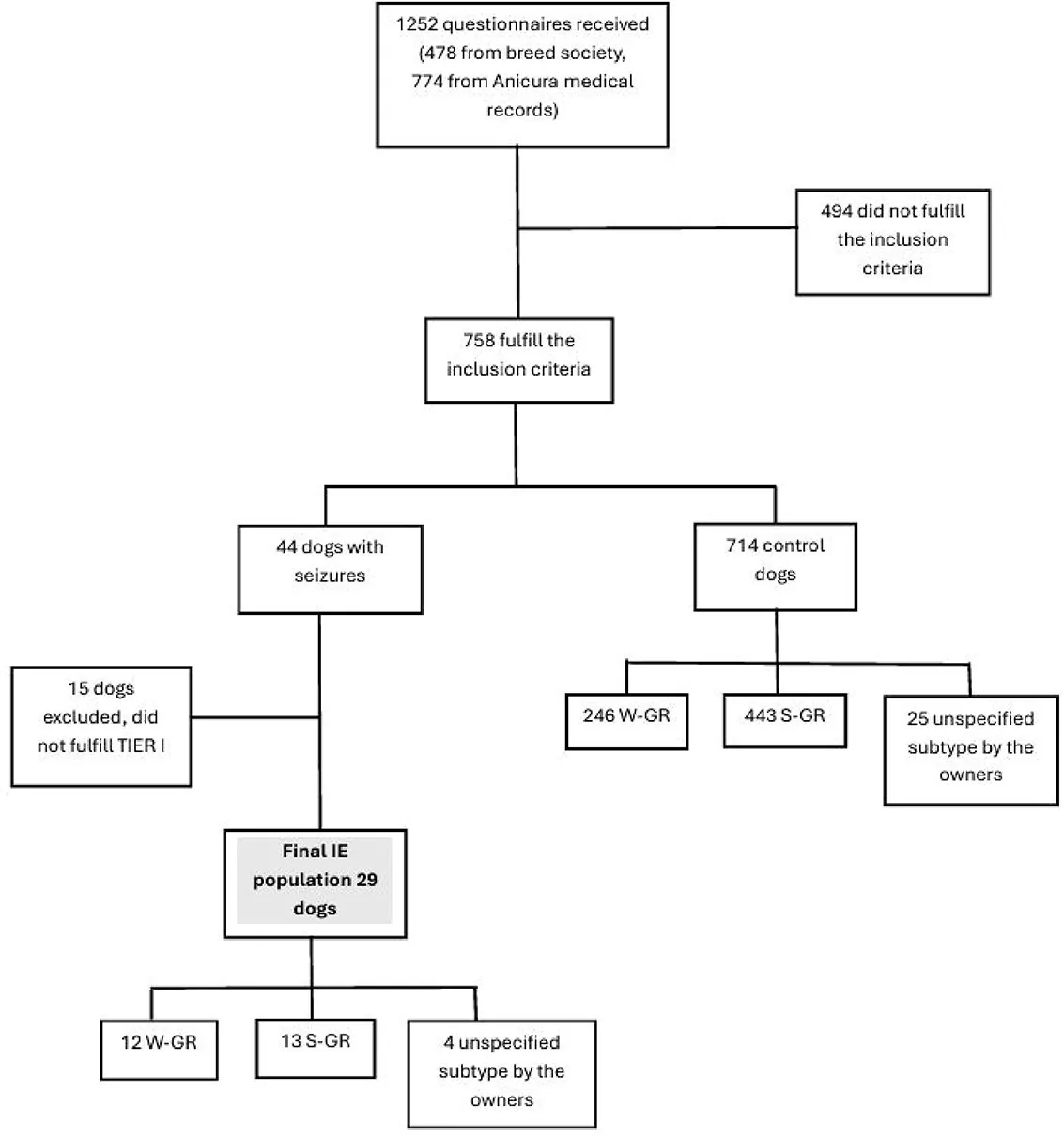
Results of the recruitment process. IE, idiopathic epilepsy; S-GR, show Golden Retriever; W-GR, working Golden Retriever.

**Table 1:** Seizure characteristics in Golden Retrievers with idiopathic epilepsy.

	Female (*n* = 9)	Male (*n* = 20)	*P*-value	S-GR (*n* = 17)	W-GR (*n* = 12)	*P*-value
**Median age at onset**	39 months (IQR 27)	38 months (IQR 37)	.981	39 months (IQR 38)	40.5 (IQR 42)	.55
**Neutered**	3 (33.3%)	9 (45%)	.694	8 (47%)	4 (33.3%)	.70
**Primary generalized tonic–clonic seizures**	7 (77.8%)	16 (80%)	1.000	14 (82.3%)	9 (75%)	.67
**Focal seizures without generalization**	-	1 (5%)	1.000	-	1 (8.3%)	.41
**Focal seizures with generalization**	2 (22.2%)	3 (15%)	.633	3 (17.6%)	2 (16.7%)	1.00
**Cluster seizures**	3 (33.3%)	11 (55%)	.427	9 (52.9%)	5 (41.7%)	.71
**Status epilepticus**	1 (11.1%)	2 (10%)	1.000	1 (5.8%)	2 (16.7%)	.54
**Seizures during rest**	6 (66.7%)	15 (75%)	.675	12 (70.6%)	9 (75%)	1.00
**Prodrome**	2 (22.2%)	13 (65%)	.050	7 (41.2%)	8 (66.7%)	.26
**Post-ictal phase**	9 (100%)	19 (95%)	1.000	17 (100%)	11 (91.7%)	.41
**Change of behavior after onset of IE**	2 (22.2%)	7 (35%)	.669	4 (23.5%)	5 (41.7%)	.68
**Treatment response (>50% reduction)**	3 (33%)	11 (55%)	.363	8 (47%)	6 (50%)	.40

Abbreviations: S-GR, show Golden Retriever; W-GR, working Golden Retriever.

### Prevalence and risk factors for IE in GRs

The prevalence of IE in GRs in the total cohort was 3.9% (29/743), with seizure prevalence not significantly different between the 2 subtypes of GRs (W-GR, 4.9%; S-GR, 2.9%; *P* = .3).

A significant association was found between IE and sex (male, female; *P* = .03), sex and neuter status (*P* = .04), feed composition (*P* = .002), and gastrointestinal signs (*P* = .01). The estimates on the logistic scale and odds ratios (OR) are shown in Table 2. Males had a higher risk for epilepsy (*P* = .04; OR = 2.38) than females. Neutered male dogs had a higher risk for epilepsy than intact males, but it was not significant at the 5% level (*P* = .05; OR = 2.57). Neutered male dogs had a significantly higher risk than intact females (*P* = .01; OR = 4.59). Gastrointestinal signs increased the risk of epilepsy (*P* = .01; OR = 3.89), and cereal-free feed posed a higher risk for epilepsy (*P* = .002; OR = 4.91). No association was found between IE and dermatologic signs (*P* = .45). Also, feed composition was not associated with either gastrointestinal signs (*P* = .7) or dermatologic signs (*P* = .34).

**Table 2:** Odds ratios, estimates, standard errors (SE), and *P*-values (both for individual levels and for main effect (bold)) on the logit scale from a logistic regression model with idiopathic epilepsy as the response variable, with sex, feed composition, and gastrointestinal problems tested as explanatory variables in the model.

Item	Odds ratio	Estimate	SE	*P*-value
**Intercept**		−4.6945	0.5022	2.00
**Sex**				**.04**
** Intact male**	1.79	0.5803	0.5031	.25
** Neutered male**	4.59	1.5244	0.5435	.01
** Intact female**	1.0	0		
** Neutered female**	1.13	0.1201	0.8326	.89
**Feed**				**.002**
** Cereal-free**	4.91	1.5914	0.486	.001
** Other**	1.00	0		
**Gastrointestinal signs**				**.01**
** Yes**	3.89	1.3589	0.4674	.004
** No**	1.0	0		

### Characterization of seizures in GRs with IE

Seizure characteristics of GRs with IE are presented in Table 1. Golden retrievers in the IE group experienced their first seizure between 7 months and 6 years of age, with a median age at onset of 36 months.

Generalized tonic–clonic seizures were the most common type of seizures; 6 owners reported focal seizures with or without secondary generalization. Seizures had a duration of 20 seconds to 5 minutes, with 16/29 (55.2%) of the seizures lasting <2 minutes. Seizures during rest or sleep were reported in 21/29 (72.4%) dogs. Eight dogs experienced seizures during activity, and 5/29 (17.2%) dogs had experienced at least 1 seizure during feeding.

Seizure episodes typically were characterized by lateral recumbency, pedal movements, hypertonicity of the neck and limbs, and hypersalivation. Cluster seizures were reported in 14/29 (48.2%) of the dogs; 11/14 (78.5%) of these were males (4 neutered). Status epilepticus was reported in 3 dogs, 2 males, and 1 female. Seizure characteristics were not significantly different in the 2 subtypes of GRs (S-GR and W-GR; Table 1).

### Prevalence and characterization of signs during prodrome and post-ictal period, and potential triggers

Clinical signs during prodrome were recognized by 15/29 (51.7%) owners, most frequently described as contact seeking and restlessness. Owners reported the prodrome to be <30 minutes (13/15, 86.7%), 12-24 hours (1/15, 6.7%) and 1-2 days (1/15, 6.7%).

Post-ictal signs were recognized by 28/29 (96.6%) owners. Aggression during the post-ictal phase was reported in 9/29 (31%) dogs (7 males and 2 females), in addition to contact seeking and restlessness. Owners reported the post-ictal period to be <30 minutes (17/26, 65.4%), 30-60 minutes (5/26, 19.2%), 1-2 hours (2/26, 7.7%) or 2-6 hours (2/26, 7.7%). The post-ictal phase for the dogs with aggression was <30 minutes for 5/9 (55.5%) dogs and >30 minutes for 4/9 (44.4%) dogs.

A suspected trigger for the seizures was reported by 14/29 (48.3%) owners, with stress (not characterized by the owner) being most common (7/14, 50%). Suspected triggers for IE in GRs are presented in Table 3.

**Table 3:** Suspected triggers for seizures in Golden Retrievers.

	GR with IE
**Stress (unspecified)**	7/14 (50%)
**Gastrointestinal signs**	12/21 (57.1%)
**Eating**	5/14 (35.7%)
**Sleeping away from home**	3/14 (21.4%)
**Sexual excitement**	2/14 (14.3%)
**Hot weather**	2/14 (14.3%)
**Change of routines**	1/14 (7.1%)
**Cold weather**	0/14 (0%)
**Doorbell/visitors at home**	0/14 (0%)
**Visit to the vet**	0/14 (0%)

Abbreviations: GR, Golden Retrievers; IE, idiopathic epilepsy.

### Administration of antiseizure medications

Treatment with 1 < (*n* = 18) or multiple (*n* = 5) antiseizure medications (ASM) was reported by 23 of 29 (79.3%) owners of GRs with IE. The most commonly administered ASM was phenobarbital (21/23, 91.3%) followed by potassium bromide (5/23, 21.7%), imepitoin (1/23, 4.3%), levetiracetam (1/23, 4.3%) and gabapentin (1/23, 4.3%). The combination of phenobarbital and potassium bromide was the most commonly used polytherapy combination (4/5, 80%).

Adverse effects associated with treatment using ASM were reported by 78.2% (18/23) of the owners. The most commonly reported adverse effects were polyphagia (7/18, 38.9%), ataxia (7/18, 38.9%), fatigue (7/18, 38.9%) and polydipsia (6/18, 33.3%).

### Outcome

Seizure freedom or a decrease in seizure frequency by at least 50%, since the start of ASM treatment, was reported in 14/23 (60.9%) of GRs treated with 1 or 2 ASM. Five (21.7%) of the GRs were refractory to treatment and showed no decrease in seizure frequency after initiation of 1 or 2 ASM.

Three GRs in the IE group were euthanized during the study period (including the entire time the questionnaire was accessible online and 3 months after its closure). Two of these GRs were young dogs (1 male, 36 months old and 1 female, 42 months old) with CS that did not respond to treatment with more than 1 ASM. Both dogs were euthanized because of their IE. The third dog was euthanized for unrelated causes.

## Discussion

Our prospective study provides important insights into the prevalence and clinical characteristics, as well as the occurrence of potential risk factors or co-morbidities, associated with IE in GRs. The prevalence of IE was 3.9%, markedly higher than the reported prevalence in the general dog population (0.62%), supporting a breed predisposition and genetic background for IE in GR as previously proposed.^2^^,^^3^ This finding confirms that IE represents a clinically important disorder in GR. Prevalence of IE in GR has not been reported in earlier studies, but can be estimated as 1% based on data reported in 1 study (19 affected cases out of 1893 included GR) from United Kingdom.^3^ The higher prevalence of IE in our population of GR compared with the population of GR in United Kingdom could be associated with genetic differences in the same breed between countries or differences in study design.

A primary aim of our study was to investigate potential risk factors related to breed subtype and sex. Although W-GRs were hypothesized to be more susceptible to IE because of their heightened energy levels and presumed increased sensitivity to stressors, no significant difference in prevalence or seizure characteristics was detected between W-GRs and S-GRs. This finding is noteworthy because epilepsy is relatively common in certain other working dog breeds such as Border Collies and Australian Shepherds, and suggests that, despite behavioral and breeding differences between the GR subtypes, IE susceptibility and phenotype appear consistent across the breed.^2^^,^^8^

### Signalment and risk factors

A significant finding was the overrepresentation of males in the IE group, and males showed a higher risk of IE in the logistic regression analysis. There also was a tendency toward an association between neutered males and IE, with 9/20 (45%) neutered males in the IE group versus 79/338 (23%) neutered males in the control group. Although causality cannot be determined from this observational data, these findings are consistent with previous reports suggesting sex- and hormone-related differences in seizure susceptibility.^4^^,^^26^ Sex hormones have an impact on seizures and epilepsy in both humans and dogs and the associations between neuter status and IE in dogs have been studied before with contradictory results.^3^^,^^26–28^ Two previous studies showed a positive association between neuter status and epilepsy,^27^^,^^28^ whereas another study found no association between neuter status and epilepsy in their population.^3^ None of these studies included information about the timing of neutering in relation to first seizure.^3^^,^^27^^,^^28^ All neutered males in the IE group in our study were neutered before their first seizure, indicating that sex hormones potentially could have a protective effect for seizures. Progesterone and testosterone seem to have anti-convulsive effects in humans and animal models, but the role of testosterone in the development of seizures in dogs is not clear.^26^ There is also a possibility that neutering results in an imbalance of testosterone and cortisol leading to increased anxiety and stress, which could be a trigger for IE.^29^

Stress (unspecified by owners) together with “sleeping away from home” and sexual excitement, 2 conditions that also could cause an intrinsic stress reaction with release of stress hormones, were the most commonly reported triggers for epileptic seizures in our study.

These results are in agreement with previous studies in which stress has been identified as a trigger for seizures in both dogs and humans with epileptic seizures.^10–16^ Hormones released during stressful conditions affect the brain in an excitatory manner and can decrease the seizure threshold.^12^ Previous studies on seizure-precipitating factors have primarily focused on exogenous influences.^11^ However, endogenous stress, such as from chronic conditions such as inflammatory gastrointestinal or dermatologic diseases, also might increase stress. These conditions can increase cortisol concentrations and circulating concentrations of cytokines or other inflammatory mediators, thereby potentially promoting the progression of epileptic seizures.^30–33^ Chronic stress therefore might contribute as a factor underlying the association between IE and gastrointestinal signs in GRs in our study.

Five dogs in our study experienced at least 1 seizure while eating. Epileptic seizures triggered by eating are rarely reported in dogs, but in 1 study, 10 dogs with seizures triggered by eating were reported, with 4 of these dogs being retrievers.^34^ Golden Retrievers are known to have strong appetite, and it is possible that the feeding routine causes a stress reaction that might trigger epileptic seizures.

### Gastrointestinal and dermatologic signs and feed composition

A noteworthy and novel observation in our study was the significantly increased prevalence of gastrointestinal signs in the IE group compared with controls. This association between IE and gastrointestinal signs supports the emerging hypothesis of a gut-brain axis involvement in epilepsy.^35–37^ It suggests that underlying gastrointestinal dysfunction might either contribute to or co-occur with neurologic disease in dogs. Previous studies have shown a difference in the composition of the gut microbiome between dogs with and without IE and between dogs with IE responsive to treatment with ASM and dogs with refractory IE.^35^^,^^38^ Further research into the association between IE and gastrointestinal disease across other dog breeds with IE is warranted and potentially could suggest new treatment strategies.

A significant association between IE and lack of cereals in the diet was noted in our population, but with no association between dogs fed a cereal-free diet and gastrointestinal signs. Cereal-free diets have gained in popularity over the last few years, and this change might cause unknown effects that promote progression of epileptic seizures in dogs. On the other hand, it is possible that cereal-free diets provide positive effects to dogs with IE, similar to the effect evidenced in gluten-associated paroxysmal dyskinesia.^39^^,^^40^ In our study, the temporal relationship between the initiation of a cereal-free diet and seizure onset was unknown, and it is therefore possible that the cereal-free diet could be a component of the dogs’ treatment plans to address IE, thereby limiting the strength of conclusions that can be drawn from this association.

Dermatologic signs were not associated with IE in our study. Dermatologic signs as endogenous stressors for IE have not been described previously in dogs, but because humans with atopic dermatitis have a higher risk of epilepsy compared with humans without atopic dermatitis (presumably because of chronic inflammation or shared genetic factors between the diseases), we wanted to test the hypothesis on GR with IE.^19^^,^^22^^,^^23^ However, a similar association could not be confirmed in our population of GRs. This lack of association could be attributed to an insufficient sample size of dogs suffering from IE, underdiagnosis of dermatologic signs by the owners of dogs with IE, or because dermatologic signs in dogs are less prone to cause a release of endogenous stress hormones that could promote progression of epileptic seizures than are gastrointestinal signs.

### Seizure characteristics

The clinical presentation of seizures in our cohort was similar to previously described IE in GRs, with GTCS being the most reported type.^4^^,^^17^^,^^18^ Notably, the majority of seizures occurred during rest, a pattern often seen in IE. Cluster seizures were common, affecting almost half of the dogs, and these were especially prevalent in males. A difference in occurrence of CS between the 2 subtypes of GRs was not detected. The occurrence of CS has implications for both prognosis and management, because CS are associated with increased risk of mortality.^41^ Epileptic dogs with CS are more likely to have drug-resistant epilepsy, which probably contributes to a higher risk of euthanasia and shorter survival time.^42^ Cluster seizures were not reported in 2 earlier Swiss studies of IE in GRs,^4^^,^^17^ but was a frequent finding (65.6%) in a Scandinavian referral population of GRs recently studied.^18^ In a previous study comparing quality of life (QoL) among breeds affected by IE, GRs were described as having a more favorable outcome than breeds such as Border Collies and Australian Shepherds, which are known for a high prevalence of CS.^43^ This observation contrasts with our findings and the findings of a previous study, in which a high prevalence of CS was observed.^18^ These discrepancies, leading to variations in seizure characteristics, might reflect differences in genetic background of GRs with IE in Denmark and Sweden compared with previously studied European populations of GR with IE,^4^^,^^17^^,^^43^ or differences in study design*.*

### Clinical signs during prodrome and the post-ictal phase

The most commonly reported clinical signs during prodrome and post-ictal phase were restlessness and contact seeking, corresponding to what has previously been described in other breeds with IE and in earlier studies of IE in GRs.^4^^,^^7^^,^^8^^,^^44^ Clinical signs during prodrome were significantly more frequent in male dogs. Although this difference was statistically significant, the underlying reason for the sex difference remains unclear. Potential causes include a more severe expression of IE in males or possibly more attentive owners of the male dogs in this population.

Post-ictal aggression, previously documented in GRs with IE,^4^^,^^18^ was common in our population. The occurrence of post-ictal aggression (31%) was substantially higher than that reported previously (approximately 8.5%), including a larger population of dogs with IE,^44^ but identical to that of another study.^18^^,^^44^ The post-ictal phase has been shown to have a significant impact on QoL in both dogs and their owners.^44^ Aggression in this phase is a contributing factor to decreased owner QoL and, together with the high frequency of CS, might contribute to the risk of euthanasia in GRs with IE. Post-ictal aggression should be considered when deciding whether to put the dog on ASM, because post-ictal aggression might represent a threat to family members. The high frequency of clinical signs during the prodrome and post-ictal phase suggests that owner education (ie, helping owners understand when a seizure is developing and giving them a treatment plan when the dog presents with a prodrome) could improve treatment of IE and potentially decrease the risk for CS in dogs prone to CS or SE.

### Treatment response and outcome

Although most of the treated dogs achieved seizure control, nearly a quarter showed no response, emphasizing the challenge of managing IE. Half of the dogs with refractory seizures did not experience CS or SE, emphasizing the complexity of the disease and the difficulties in predicting which patients are likely to respond well to treatment and which patients will show poor response. The euthanasia of 2 young GRs because of poor seizure control indicates the severity of the disease in some individuals.

### Study limitations

Our study shares the inherent limitations of a questionnaire-based study, including the potential for inaccurate or biased responses. Additionally, limitations regarding the depth and detail of responses provided exist because of the use of closed-ended questions. Additional limitations include the relatively short period during which the questionnaire was available on the Netigate platform. We also provided the owners with a video of a GR with a GTCS, which potentially could have caused under-reporting of dogs with focal seizures. The IE group was rather small, and most of the dogs did not undergo magnetic resonance imaging or cerebrospinal fluid analysis. The control group, however, was large and provided a robust reference population for comparison.

## Conclusions

Idiopathic epilepsy in Swedish GRs is considerably more prevalent than in the general dog population and is characterized by GTCS, frequent CS and common post-ictal aggression. Breed subtype was not associated with IE, whereas male sex and gastrointestinal signs were significant associations. The observed link between IE and gastrointestinal signs, and the tendency toward an increased risk in neutered males, were novel findings.

## Supplementary Material

Supplementary_video_IE_Golden_aalag230

## Off-label antimicrobial declaration

Authors declare no off-label use of antimicrobials.

## Institutional animal care and use committee or other approval declaration

Authors declare no institutional animal care and use committee or other approval was needed.

## Appendix

### Material and methods

#### Recruitment process

The recruitment process included multiple channels to ensure broad participation including announcements through the Swedish Breed Society for Golden Retrievers via their website, social media and membership magazine. The announcement also was disseminated in various social media groups focused on GRs and epileptic dogs. In addition, direct invitations were sent to owners of GRs born between 2010 and 2023, identified from Anicura Sweden’s medical records. Email invitations were sent to owners with registered email addresses, regardless of the dog’s medical history.

Owners with more than 1 GR were requested to complete the survey separately for each dog. The survey link was reposted twice on social media platforms, whereas the email invitation was sent once. The entire questionnaire was presented in Swedish.

The questionnaire included 3 main sections: signalment (1), general health (2), and seizure characteristics (3).

The signalment section included general questions related to sex, neuter status, breed type (S-GR or W-GR), and living conditions (single or multi-dog household, amount of daily exercise, type of food).

The general health section included questions related to the dogs´ general health and specific questions related to the presence of clinical signs indicating gastrointestinal or dermatologic disease. The questions related to gastrointestinal signs were adapted from a previous study.^45^

The seizure characteristics section included questions related to age at seizure onset, seizure type (generalized, focal, or a combination), occurrence of cluster seizures, status epilepticus or both, owner-identified triggers, presence of pre- and post-ictal signs, administration of anti-seizure medication (ASM), response to treatment, and adverse effects of ASM use.

#### Statistical analysis

The descriptive statistical analyses were performed using a commercially available statistical software program (JMP Pro v. 11.2.0-17.2.0), Cary, NY, USA. Data was analyzed using descriptive and inferential statistics. Descriptive statistics are reported for continuous variables using mean (SD) for approximately normally distributed values, and median (IQR) for non-normal data. Differences in proportions concerning dog characteristic variables (male/female, S-GR/W-GR) and variables related to seizure characteristics (primary generalized seizures, focal seizures without generalization, focal seizures with generalization, cluster seizures, status epilepticus, seizures during rest, pre-ictal phase, post-ictal phase, changes of behavior after onset of seizures, treatment response) and neuter status, were tested using Chi-squared-tests or Fisher’s exact test when small sample sizes were compared. Differences in proportions concerning dog characteristics (age at onset of seizures) and sex (male/female) were performed using the Wilcoxon test after testing for normality using the Shapiro–Wilk test. The statistical significance level was set at *P* < 0.05 for all tests performed.

We fitted a logistic regression model to estimate the effect of potential risk factors on the probability of IE. Let p be the probability of epilepsy given the factors in the model, then:

\begin{align*} \mathrm{logit}\left(\mathrm{p}\right)=\,&\log \left[\mathrm{p}/\left(1-\mathrm{p}\right)\right]=\mathrm{\mu} +\mathrm{se}{\mathrm{x}}_{\mathrm{i}}+\mathrm{cerea}{\mathrm{l}}_{\mathrm{j}}+\mathrm{gas}{\mathrm{t}}_{\mathrm{k}}+\mathrm{typ}{\mathrm{e}}_{\mathrm{l}}\nonumber\\&+\mathrm{e}{\mathrm{x}}_{\mathrm{m}}+\mathrm{h}{\mathrm{h}}_{\mathrm{n}}+\mathrm{com}{\mathrm{p}}_{\mathrm{o}}+\mathrm{der}{\mathrm{m}}_{\mathrm{g}}+\mathrm{fee}{\mathrm{d}}_{\mathrm{s}} \end{align*}

where μ was the overall mean (intercept), sex_i_ was the fixed effect of birth sex and neuter status combination (intact male, neutered male, intact female, neutered female) or only sex (male, female), feed_j_ was the fixed effect of feed composition (cereal-free, other, missing information), gast_k_ was the fixed effect of gastrointestinal signs (yes, no, missing information), type_l_ was the fixed effect of subtype (working, show, dual purpose or unknown), ex_m_ was the fixed effect of daily exercise level (< 30 minutes, 30-60 minutes, 1-2 hours, 2-3 hoursoursours, and > 3 h), hh_n_ was the fixed effect of single dog household vs. multi-dog household, comp_o_ was the fixed effect of participation in competitions (obedience, hunting, agility etc., yes or no), derm_g_ was the fixed effect of dermatologic signs (yes or no), and feed_s_ was the fixed effect of type of feed (only dried feed or not). The levels for missing information and for dual purpose or unknown were not of interest per se, but were included to avoid losing observations.

The model was fitted using function glm (family = binomial) in R.^45^ Factors were tested for significance using a type III test (ANOVA in the package car); the factor with the lowest p-value was removed repeatedly until only factors with p-values < 0.05 remained (backward elimination).

## Abbreviations

ASM: antiseizure medication
CS: cluster seizures
GR: Golden retrievers
GTCS: generalized tonic–clonic seizures
IE: idiopathic epilepsy
IVETF: International Veterinary Epilepsy Task Force
IQR: interquartile range
MRI: magnetic resonance imaging
MQ: main question
OR: odds ratio
QoL: quality of life
SD: standard deviation
SE: status epilepticus
S-GR: show Golden Retriever
W-GR: working Golden Retriever
